# Fine-Scale Bacterial Beta Diversity within a Complex Ecosystem (Zodletone Spring, OK, USA): The Role of the Rare Biosphere

**DOI:** 10.1371/journal.pone.0012414

**Published:** 2010-08-26

**Authors:** Noha H. Youssef, M. B. Couger, Mostafa S. Elshahed

**Affiliations:** Department of Microbiology and Molecular Genetics, Oklahoma State University, Stillwater, Oklahoma, United States of America; University of Hyderabad, India

## Abstract

**Background:**

The adaptation of pyrosequencing technologies for use in culture-independent diversity surveys allowed for deeper sampling of ecosystems of interest. One extremely well suited area of interest for pyrosequencing-based diversity surveys that has received surprisingly little attention so far, is examining fine scale (e.g. micrometer to millimeter) beta diversity in complex microbial ecosystems.

**Methodology/Principal Findings:**

We examined the patterns of fine scale Beta diversity in four adjacent sediment samples (1mm apart) from the source of an anaerobic sulfide and sulfur rich spring (Zodletone spring) in southwestern Oklahoma, USA. Using pyrosequencing, a total of 292,130 16S rRNA gene sequences were obtained. The beta diversity patterns within the four datasets were examined using various qualitative and quantitative similarity indices. Low levels of Beta diversity (high similarity indices) were observed between the four samples at the phylum-level. However, at a putative species (OTU_0.03_) level, higher levels of beta diversity (lower similarity indices) were observed. Further examination of beta diversity patterns within dominant and rare members of the community indicated that at the putative species level, beta diversity is much higher within rare members of the community. Finally, sub-classification of rare members of Zodletone spring community based on patterns of novelty and uniqueness, and further examination of fine scale beta diversity of each of these subgroups indicated that members of the community that are unique, but non novel showed the highest beta diversity within these subgroups of the rare biosphere.

**Conclusions/Significance:**

The results demonstrate the occurrence of high inter-sample diversity within seemingly identical samples from a complex habitat. We reason that such unexpected diversity should be taken into consideration when exploring gamma diversity of various ecosystems, as well as planning for sequencing-intensive metagenomic surveys of highly complex ecosystems.

## Introduction

The adaptation of pyrosequencing technologies [1] for use in culture-independent diversity surveys allowed for deeper sampling of ecosystems of interest. During the last few years, pyrosequencing surveys of prokaryotic diversity has been instrumental for more accurate exploration of species richness, evenness, and total diversity in highly diverse ecosystems [2], [3], [4], [5], detailed phylogenetic description of microbial community structure at the phylum, class, and order levels [6], [7], gaining access to members of the community present in low abundance [8], [9], and for zooming in on the diversity of specific phylogenetic lineages of interest [4], [10]. In addition to phylogenetic and statistical examination of microbial diversity in a single sample (i.e. alpha diversity), pyrosequencing has been used recently for comparative diversity studies, in which various aspects of microbial community composition is compared between multiple (few to hundreds) samples. The use of a bar-coding strategy [11], in which unique sequence identifiers to PCR primers allowed for pooling and simultaneous sequencing of a large number of distinct samples in a single pyrosequencing run followed by bioinformatic sorting of sequences, allowed for comparative diversity studies to be conducted across hundreds of samples simultaneously in a single sequencing run. Comparative studies have been used in multiple habitats to: correlate environmental variables to observed microbial community structure and composition [12], investigate the effect of natural and anthropogenic disturbances on a specific ecosystem, delineate intra and inter variations in microbial community patterns amongst skin and internal organs of healthy and diseased subjects [13], [14], [15], [16], and conduct time depth, and spatial surveys of microbial communities in marine ecosystems [6], [9], [17], [18].

One potential area of interest that is extremely well suited for pyrosequencing-based diversity surveys that has received surprisingly little attention so far, is examining fine scale (e.g. micrometer to millimeter) beta diversity in complex microbial ecosystems. Fine scale beta diversity in seemingly identical (replicate) samples from complex ecosystems is an extremely important, yet poorly-understood issue that could have various implications on estimates of overall species diversity within an ecosystem (i.e. Gamma diversity), on understanding and gauging level of ecosystem resiliency and capability to respond to natural and anthropogenic environmental disturbances, on patterns of microbial dispersal and immigration in an ecosystem, as well as on planning for metagenomic surveys of highly diverse ecosystems. We here present our effort in gauging the level of fine scale beta diversity in a complex highly diverse ecosystem (Zodletone spring, Oklahoma USA). The source of Zodletone spring is composed of highly sulfidic sediments overlaid by sulfide-laden water, and are largely undisturbed and unexposed to atmospheric air. The anaerobic nature of the spring prevents any plant growth that might affect bacterial patterns. As such, replica fine scale sampling from such sediments could be regarded as a true measure of spatial difference between samples exposed to identical geochemical and environmental parameters, rather than an exploration of the effect of environmental variations on microbial communities. We used these replica samples to demonstrate that significant levels of beta diversity exist on a mm-scale in Zodletone spring, that these changes are truly spatial and could not be explained by sample size inadequacy, and that rare members of the microbial community are primarily responsible for such levels of diversity.

## Materials and Methods

### Site description and sampling

Sediments were collected from the source area of Zodletone spring, a sulfide- and sulfur-rich spring in southwestern Oklahoma in May 2009. The source of the spring is a contained area (1 m^2^) in which anaerobic, biomass-laden, and sulfide-rich black viscous sediments are covered by an anoxic, sulfide-rich (8.4 mM) 40-cm water column (Figure S1a). Detailed description of the spring geochemistry has been described elsewhere [19], [20].

Previous exploration of the bacterial diversity, *in-situ* activities, and prevalent geochemical conditions at the spring suggested that multiple factors contribute to the high level of bacterial alpha diversity reported at the source sediments (66 OTUs identified within a library of 116 clones from the spring source) [20]. The prevalence of strict anoxic conditions, coupled to the abundance of multiple sulfur species (sulfide, elemental sulfur, thiosulfate, and sulfate), and direct exposure to sunlight result in a thriving chemolithotrophic, anoxygenic phototrophic, as well as sulfate-, thiosulfate- and sulfur-reducing and disproportionating communities involved in sulfur cycling in the spring [20]. As well, the abundance of short chain hydrocarbons results in the presence of thriving hetertrophic community [20], [21].

While the microbial community at Zodletone spring source sediments could be affected by depth due to subtle attenuation in light-penetration and intensity by depth, and the subsequent alteration of phototrophic-driven sulfur cycling patterns [22], we reason that no significant changes in geochemical conditions would be encountered between adjacent samples at the same depth (e.g. surface sediments from the same height in the source). The source sediments at the surface are quite immobile and are overlaid by sulfidic water that maintains near constant temperature throughout the year. Moreover, due to the anoxic, sulfidic conditions at the spring source, no plant growth, or plant root structures are present in the spring source, which would lead to drastic nutrient and colonization-driven variations in bacterial abundance and composition, an issue often encountered in soil and rhizosphere studies. As such, we believe that source sediments from Zodletone spring are ideal for examining fine scale beta diversity in natural ecosystems.

Sampling was conducted to obtain four samples (quadrants) that are 1mm apart. The sampling technique is shown in figure S1b. Briefly, a sampling grid with 1-in^2^ squares, and grid of 1 mm was used to capture anaerobic sediment from the source of the spring. Sediments were stored undisturbed on ice until transferred to the lab where 4 samples (1 g each) from the grid corners (Figure S1b), obtained from the same depth were used for DNA extraction.

### DNA extraction, PCR amplification and pyrosequencing

DNA was extracted from each of the 4 samples using the FastDNA Spin kit for Soil (MPBiomedicals, Solon, OH). Variable regions V1 and V2 of the 16S rRNA gene were then amplified using primers for bar-coded mass parallel sequencing using the FLX technology. The forward primer was constructed by adding FLX adaptor A (GCCTCCCTCGCGCCATCAG) to the 27F primer sequence (AGAGTTTGATCCTGGCTCAG). The reverse primer was constructed by adding FLX adaptor B (GCCTTGCCAGCCCGCTCAGT) to the 338R primer sequence (GCTGCCTCCCGTAGGAGT). PCRs were conducted in 100µl volume. The reaction contained 4 µl of the extracted DNA, 1× PCR buffer (Promega, Madison, WI), 2.5 mM MgSO4, 0.2 mM dNTPs mixture, 0.5U of the GoTaq flexi DNA polymerase (Promega, Madison, WI), and 10µM each of the forward and the reverse primers. PCR was carried out according to the following protocol; initial denaturation at 95°C for 5 minutes, followed by 35 cycles of denaturation at 95°C for 45 sec, annealing at 52°C for 45 sec, and elongation at 72°C for 30 sec. A final elongation step at 72°C for 5 minutes was included. PCR products for each sample were combined and purified using a PCR cleanup kit (Invitrogen corp., Carlsbad, CA). Eleven to fifteen µg-DNA of purified PCR products were sequenced using FLX technology at the Environmental Genomics Core facility at the University of South Carolina.

### Sequence quality filtering

MOTHUR software [23] was used for most of the sequence processing and operational taxonomic unit (OTU) assignments. Raw sequence data were subjected to various assessments to eliminate poor quality sequences. An average quality score of 25 was chosen as the threshold value below which sequences were considered of poor quality and removed from the dataset. In addition, sequences that did not have the exact primer sequence, sequences that contained an ambiguous base (N), sequences having a homopolymer stretch longer than 8 bases, and sequences shorter than 80 bp were also removed from the datasets using trim.seqs command in MOTHUR. A total of 348,086 sequences were obtained from the four quadrants. After implementation of all quality control criteria described above, only 292,130 sequences (83.9%) were considered of high quality and retained for further analysis (Table S1).

### OTU identification and phylogenetic assignments

High-quality reads from each quadrant were aligned in MOTHUR platform [23] using the Greengenes alignment database available at the MOTHUR website as a template. Aligned sequences were then filtered to remove columns that corresponded to ‘.’ or ‘-’ in all sequences. Filtered alignments were then used to generate an uncorrected pair wise distance matrix using dist.seqs command in MOTHUR. To assign sequences into OTUs, a further neighbor-clustering algorithm was employed using the cluster command in MOTHUR, with 97% sequence similarity as the designated cutoff. Representative OTUs defined at this cutoff (OTU_0.03_) were then classified using Hugenholtz taxonomy scheme in Greengenes pipeline [24]. Phylum level affiliation of sequences were determined according to the classifier output, and sequences with less than 85% similarity to their closest relative in Greengenes database were considered unclassified.

### Defining rare and abundant species in Zodletone sprin

In addition to comparing beta diversity patterns between entire datasets, we also examined beta diversity relationships between both rare and abundant members of each dataset. We defined rarity of OTUs at multiple empirical cutoffs (n = 1, n≤2, n≤5, n≤10), as well as at a percentage abundance of ≤0.004%. The later percentage-based cutoff was used to compensate for the fact that one of the datasets (dataset 4) has fewer sequences than the three other datasets (Table 1). Galand et al. [9] used cutoff for a rare species as the lowest frequency of occurrence (n = 1) in their smallest dataset. This represents 0.002% in our pyrosequencing datasets. That small percentage abundance corresponds to n = 1 in our smallest dataset (quadrant 4) and n≤1.5–1.6 in our larger datasets (quadrants 1, 2, and 3). Since an abundance of 1.5–1.6 is not possible, we expanded our percentage abundance cutoff for rare species definition to 0.004%. This corresponds to n≤2 in quadrant 4 and n≤3 in the other quadrants.

**Table 1:** Number of high quality reads and OTUs_0.03_ identified for each of the samples (quadrants) studied.

Quadrant	Number of high-quality reads	Number of OTUs_0.03_
1	77361	18265
2	86240	17288
3	76693	18408
4	51836	13266

Abundant species were also defined at two empirical cutoffs: n>10 and percentage abundance >1%. The former definition includes many of the OTUs with intermediate abundance and results in the classification of all sequences in the dataset into either rare or abundant, while the later only considers sequences that are truly present in very high levels in such a diverse ecosystem as abundant sequences.

### Sub-classification of rare members of the community according to their novelty and uniqueness patterns

We further sub-classified rare OTUs based on their relationship to the more abundant members of the community, as well as according to their phylogenetic novelty (i.e. level of similarity to closest relatives in public databases), and examined beta diversity relationships amongst these subgroups of the rare biosphere. We blasted all sequences of the rare biosphere (defined as n≤5) from all 4 quadrants (84,532 sequences) against the combined abundant members (total of 3962 OTUs, 182,122 clones) using blastall function from the NCBI ftp site at ftp://ftp.ncbi.nih.gov/blast/executables/blast+/LATEST/. Those rare members that were >85% similar to an abundant OTU with alignment length of at least 100 bp were considered non-unique, non-novel (NUNN) i.e. with relatives encountered within the more abundant members of the dataset, and does not belong to a novel phylogenetic lineage, since all abundant OTUs within the dataset were readily assignable to a phylogenetic group. A total of 76,491 sequences within the rare biosphere were classified in this NUNN category. The phylogenetic affiliations of the remaining 8041 sequences, previously obtained using Greengenes classifier, were examined. Sequences with more than 85% similarity to a database relative were considered unique in our dataset but non-novel i.e. with no close relatives within the abundant members of the community, but with closely related relatives previously encountered in other ecosystems (UNN; 4237 sequences). Sequences that did not fulfill either of the above criteria were considered both unique and novel (UN; 3804 sequences). The NUNN, UNN, and UN fractions of the rare biosphere in each of the four samples were then extracted to separate files and various alpha and beta diversity comparisons were conducted between these groups.

### Beta diversity estimates and statistical comparisons

Rarefaction curves of various datasets were generated in MOTHUR using a re-sampling without replacement approach. Likelihood-ratio-Chi-squared test was conducted [25] to test for the significant difference between phyla relative abundances in the 4 quadrants studied. The χ^2^ values obtained were compared to the tabulated χ^2^ value [with 177 degrees of freedom ((m-1)x(n-1), where m is the number of phyla and n is the number of quadrants), and α value of 0.008 (equivalent to Bonferroni correction for α = 0.05 for 6 pair wise comparisons [26]] equivalent to 225.5. The probability of the result being due to chance (i.e. p-value) was calculated according to the equation 
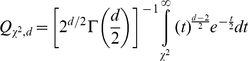
, where Q is the probability that the χ^2^ value for an experiment with d degrees of freedom is consistent with the null hypothesis. Q was evaluated using the Chi-square calculator available at http://www.fourmilab.ch/rpkp/experiments/analysis/chiCalc.html.

Multiple approaches were used to gauge beta diversity between various datasets. All of these approaches/indices are based on identifying sequences clustering into the same OTUs from different quadrants (i.e. shared OTUs). Therefore, for each beta diversity comparison conducted in this study, a joint fasta file of all sequences within datasets (or fractions of the datasets) to be compared was created, aligned, and a joint distance matrix was created in MOTHUR [23]. A shared OTUs file was also generated in MOTHUR that shows the proportion of shared versus unique sequences between the datasets compared. This file was used as a starting point for all subsequent beta diversity indices. Pair wise comparisons between two datasets were conducted using qualitative similarity indices (those that use presence/absence data) e.g. Anderberg [27], Jaccard [28], Sorensen [29], and Ochiai [30] indices, as well as quantitative indices (those that take OTUs abundance or relative abundance into consideration) e.g. Bray-Curtis [31], abundance-based Jaccard [32], abundance-based Sorensen [32], Smith theta [33], and Yue and Clayton theta [34] indices. In addition to pair wise comparison, Venn diagrams for graphical descriptions of unshared, as well as shared OTUs between two, three, or four quadrants were constructed. Non-metric multidimensional scaling plots for communities using Bray-Curtis similarity indices were created using the function metaMDS in the vegan library of R statistical package [35], [36]. The proportion of variance (r^2^) among communities was estimated from the NMDS plots by first calculating the Euclidean distance between all pairs of data points using the equation 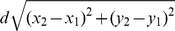 where d is the Euclidean distance between 2 points of coordinates (x_1_, y_1_) and (x_2_, y_2_) in ordination space. The Euclidean distance was then regressed on Bray-Curtis similarity indices to estimate r^2^.

### Evaluating the effect of spatial separation on β-diversity

To test whether the observed β-diversity is truly due to spatial separation, we created three random subsamples (28,000 sequences each) from our largest dataset (quadrant 2) and treated the three subsamples as separate datasets. We also randomly picked a 28,000-sequence subsample from each of the other 3 quadrants. The hypothesis is that if differences were not random (i.e. truly due to spatial separation) then the 3 subsamples drawn from the same quadrant would be more similar to each other than to subsamples from other quadrants. For all subsamples various beta diversity estimates were calculated and compared.

### Estimating inadequate sampling effect on the shared species of the rare biosphere

To evaluate whether the observed β-diversity between rare members of the biosphere is true or due to inadequate sampling, we first estimated the species richness in each quadrant using parametric model ‘mixture of 2 exponentials-mixed Poisson’ (since this model provided the best fit to our abundance data with the least standard error, highest truncation point, least χ^2^ p-value, and lowest residuals) as previously explained in detail [37], [38]. Using the obtained model parameters, we estimated the expected number of singletons in each quadrant. For each quadrant, using probability laws, we predicted the number of shared singletons when a virtual community is created by randomly drawing a number equal to the encountered number of singletons in each quadrant 4 times (corresponding to the 4 quadrants studied) with replacement from a pool of the expected number of singletons in that quadrant. We repeated that for each of the quadrants and the average predicted % of shared singletons between 2, 3, and 4 quadrants as well as the average predicted % of unshared singletons were compared to the observed % of shared and unshared singletons between the 4 quadrants studied. The hypothesis is that, if the differences observed were true and not due to inadequate sampling, the % shared OTUs in the virtual community would be more than the observed %. Similarly, the % of unshared OTUs in the virtual community would be less than the observed %. Statistical significance of differences between predicted and observed percentages was tested using a student T test.

## Results

### Community structure and phylum-level comparative beta diversity of Zodletone spring source sediments datasets

Overall, sequencing from four quadrants yielded a total of 292,130 high quality reads distributed between four quadrants derived from Zodletone samples 1 mm apart. High quality reads are available in the supporting Text S1, S2, S3 and S4. The number of sequences, and number of OTUs_0.03_ from each quadrant is depicted in Table 1. The incomplete sampling effort in spite of sequencing an average of 73,000 reads per sample (Figure S2), and the large number of distinct OTUs_0.03_ observed per quadrant (Table 1) clearly indicate a highly diverse microbial community in the spring, as previously suggested [20].

**Figure 1:**
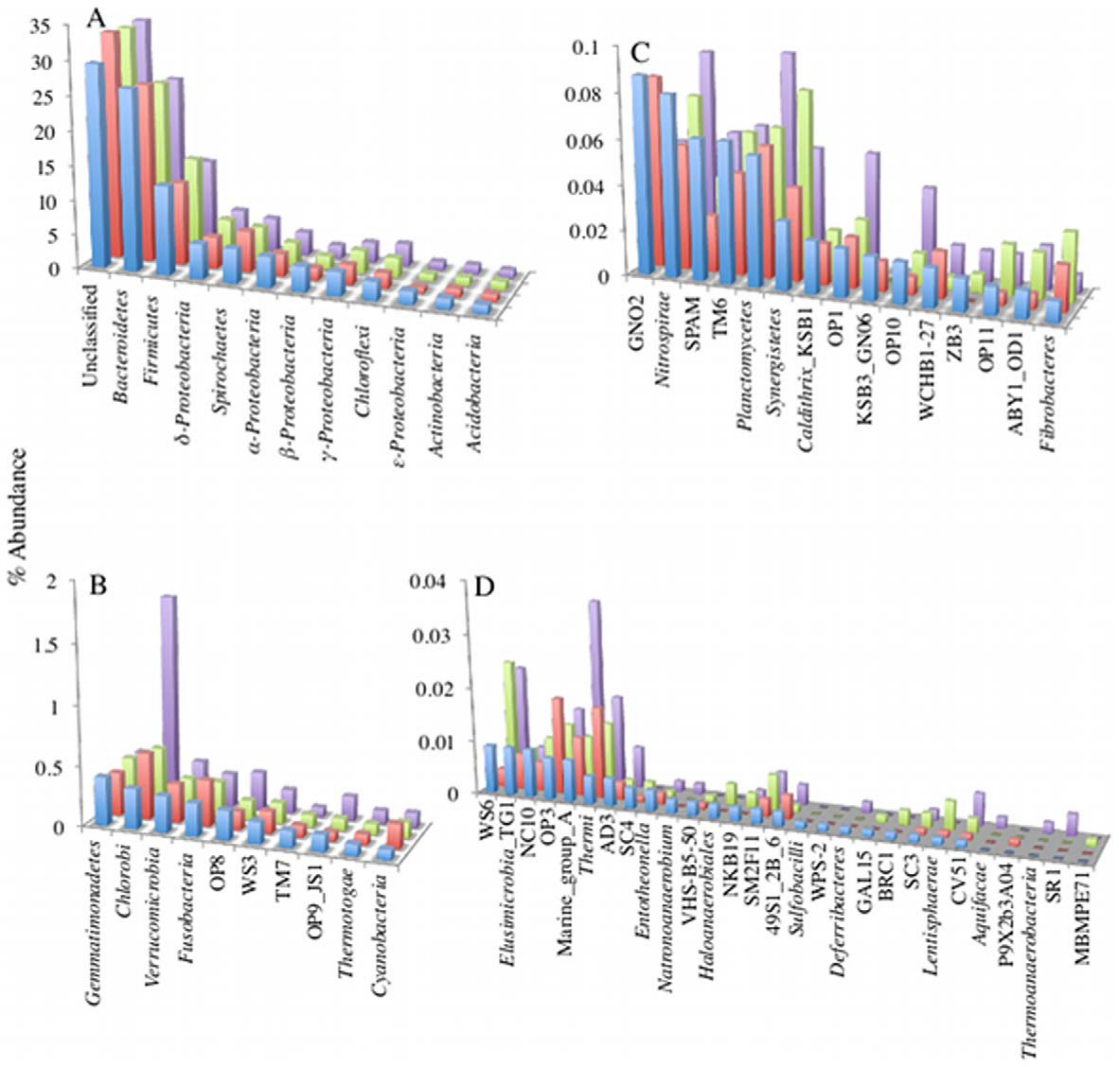
Distribution of phyla in Zodletone spring source sediment for the 4 quadrants studied. Percentage abundance is shown on the Y-axis for (A) abundant phyla with >1% abundance in each quadrant studied, (B) phyla with % abundance ranging between 0.1 and 1% in quadrant 1, (C) phyla with % abundance ranging between 0.01 and 0.1% in quadrant 1, and (D) phyla with % abundance <0.01% in quadrant 1. Color-coding is as follows: quadrant 1 (blue), quadrant 2 (red), quadrant 3 (green), and quadrant 4 (purple).

Phylum level affiliations of sequences encountered in each quadrant revealed an extremely diverse, phylum-rich bacterial community. In spite of implementing a conservative cutoff of >85% sequences similarity to closest relative in Greengenes database to assign an OTU into a specific phylum, members of 60 different bacterial phyla and candidate phyla were identified (Figure 1, Table S2). The number of phyla in Zodletone spring could potentially be higher than 60 since 32% of the sequences obtained were unclassified (Figure 1).

Comparison of the phylum level composition between the four quadrants showed highly similar communities at the phylum level. Out of the 60 phyla and candidate phyla collectively identified, 44 were identified in all four quadrants, 5 in only 3 quadrants, 6 in only 2 quadrants, and 7 in only one quadrant (Figure 1, Table S2). All phyla and candidate phyla not encountered in all four quadrants (*Aquificae*, *Natronoanaerobium*, P9X2b3A04, *Sulfobacilli*, *Thermoanaerobacteria*, WPS-2, *Deferribacteres*, GAL15, SR1, BRC1, MBMPE71, VHS-B5-50, *Haloanaerobiales*, NKB19, SC3, *Lentisphaerae*, SC4, CV51, ZB3, and KSB3_GN06) were present in extremely low (less than 27 total sequences in the 4 quadrants studied, 0.009% of the total reads) abundance. Likelihood ratio chi squared test showed no significant differences in phylum-level community composition (likelihood ratio χ2 = 33.4, p = 0.999). Further, all pair wise similarity indices at the phylum level were high (0.72–1) indicating extremely low beta diversity (Table 2).

**Table 2:** Qualitative and quantitative similarity indices at the species level for the rare and abundant members of the community (defined at specific empirical cutoffs) as well as for the totals at both the phylum and the species levels.

Cutoff*	P. level^a^	Anderberg^b^	Jaccard^b^	Ochiai^b^	Sorensen^b^	Bray-Curtis^b^	Abundance-based		Theta (Smith)^b^	Theta (Yue & Clayton)^b^
							Jaccard^b^	Sorensen^b^		
Total	OTU_0.03_	0.12±0.003	0.21±0.005	0.35±0.005	0.35±0.007	0.59±0.03	0.67±0.01	0.68±0.01	0.65±0.01	0.65±0.06
	Phylum	0.72±0.09	0.84±0.06	0.91±0.03	0.91±0.03	0.85±0.09	1±0	1±0	1±0	0.99±0.004
n = 1	OTU_0.03_	0.02±0.001	0.05±0.002	0.09±0.003	0.09±0.004	0.09±0.004	0.08±0.02	0.13±0.04	0.05±0.002	0.05±0.002
n≤2	OTU_0.03_	0.04±0.001	0.08±0.002	0.15±0.003	0.15±0.003	0.14±0.003	0.22±0.01	0.36±0.01	0.09±0.002	0.09±0.002
n≤5	OTU_0.03_	0.07±0.002	0.13±0.003	0.23±0.003	0.23±0.004	0.23±0.004	0.32±0.01	0.48±0.01	0.18±0.003	0.18±0.005
n≤10	OTU_0.03_	0.09±0.02	0.16±0.003	0.28±0.003	0.28±0.004	0.29±0.006	0.4±0.008	0.57±0.01	0.26±0.003	0.28±0.01
n>10	OTU_0.03_	0.4±0.02	0.57±0.02	0.73±0.01	0.72±0.01	0.71±0.05	0.86±0.02	0.93±0.01	0.86±0.02	0.82±0.06
a≤0.004%	OTU_0.03_	0.05±0.005	0.1±0.009	0.18±0.014	0.18±0.015	0.17±0.016	0.25±0.01	0.4±0.018	0.12±0.012	0.12±0.012
a>1%	OTU_0.03_	0.48±0.1	0.65±0.09	0.78±0.07	0.78±0.07	0.61±0.1	0.65±0.08	0.78±0.06	0.65±0.08	0.65±0.08

*The empirical cutoff used to define rare and abundant members of the community. n: corresponds to the number of clones, a: corresponds to percentage abundance. Cutoffs n = 1, n≤2, n≤5, n≤10, a≤0.004% are rare cutoffs, while n>10, and a>1% are the abundant cutoff.

a The phylogenetic level used to bin sequences into operational taxonomic units. OTU_0.03_ is the 97% cutoff for defining species.

b All similarity indices values are averages ± standard deviations of all possible (6) pair wise comparisons.

### Fine scale Beta diversity between Zodletone spring source sediment quadrants

**Figure 2:**
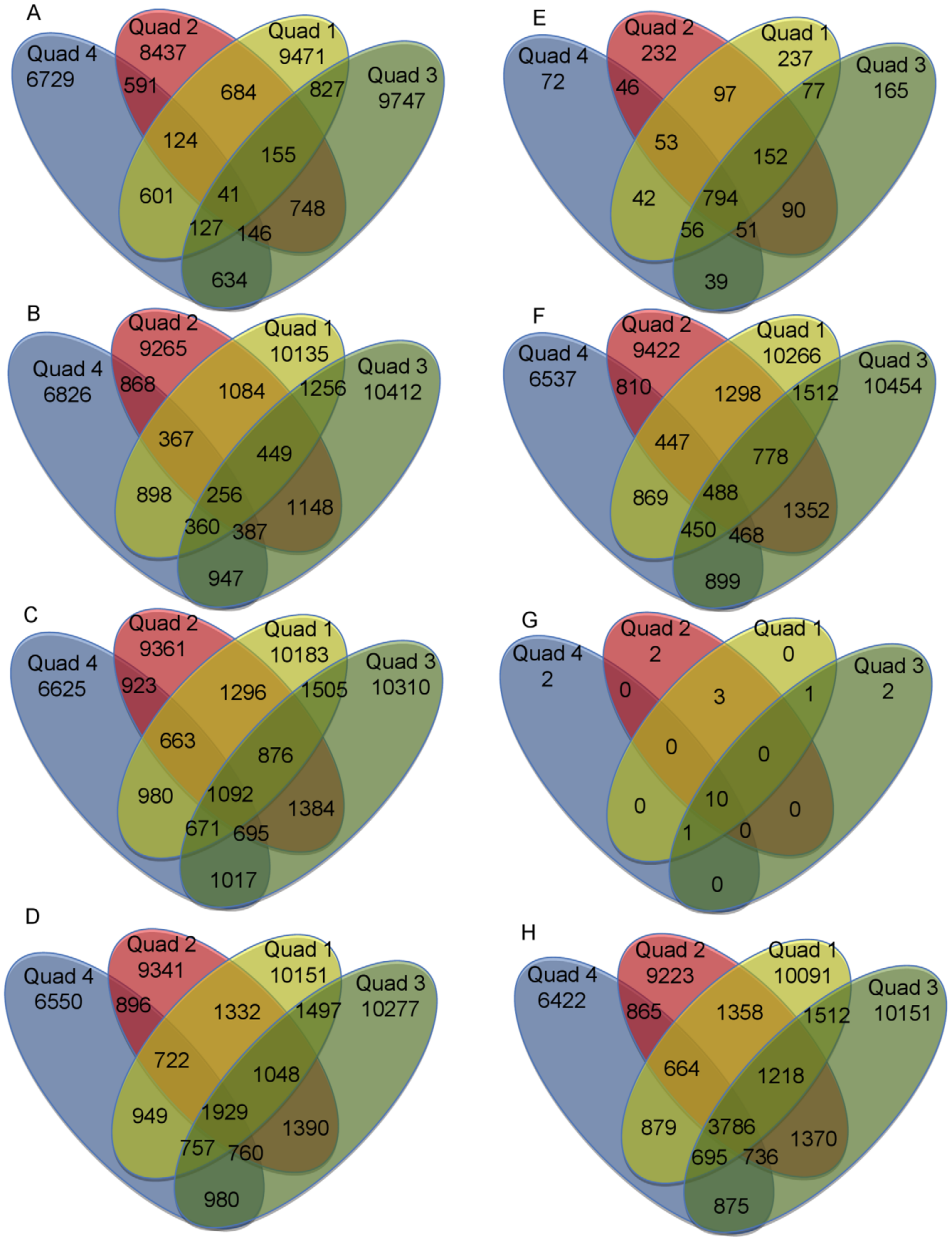
Venn diagrams showing the number of unique and shared OTUs between the 4 quadrants studied for (A) empirical cutoff (n = 1) for defining rare species, (B) empirical cutoff (n≤2) for defining rare species, (C) empirical cutoff (n≤5) for defining rare species, (D) empirical cutoff (n≤10) for defining rare species, (E) empirical cutoff (n>10) for defining abundant species, (F) empirical cutoff (% abundance ≤0.004%) for defining rare species, (G) empirical cutoff (% abundance >1%) for defining abundant species, and (H) the entire datasets. Color-coding is as follows: quadrant 1 (yellow), quadrant 2 (red), quadrant 3 (green), and quadrant 4 (blue).

Since no significant differences were observed at the phylum level between the four quadrants analyzed, beta diversity measures at OTU_0.03_ level were conducted to examine whether in such an ecosystem, high levels of beta diversity would be encountered at the species levels. Examining shared OTUs showed that, on average, 21.4±0.48% of OTUs, and 79.1±1.25% of clones are shared between any two samples (n = 6), 11.3±0.2% of the OTUs and 74.6±0.9% of clones were shared between 3 samples (n = 4), and only 7.6% of the OTUs and 72.3% of clones are shared between four samples (Figure 2H, Table S3). Further, qualitative and quantitative diversity indices between all possible pairs of samples showed values of 0.12–0.68 (Table 2) indicating higher levels of beta diversity at the species level compared to the phylum level. These results argue that while a phylum level diversity of a highly-diverse ecosystem could accurately be described by a single pyrosequencing sampling event, the identity of OTU_0.03_ could differ greatly between seemingly identical samples within an ecosystem.

### Beta diversity in rare versus abundant members of the dataset

**Figure 3:**
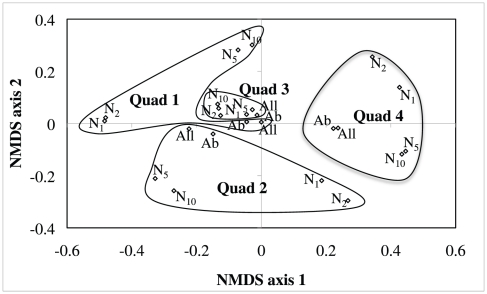
Non-metric multidimensional scaling plots of the 4 quadrants studied. Pair wise Bray-Curtis similarity indices for each of the quadrants at 4 empirical rare cutoffs (n = 1 (N_1_), n≤2 (N_2_), n≤5 (N_5_), and n≤10 (N_10_)), one empirical abundant cutoff (n>10 (Ab)), as well as for the entire datasets (All) were used to construct the plot. Grey shapes show rare and abundant members from each quadrant clustering closer together with the whole quadrant. Stress value was close to 0, and most of the variation (65–95.3%) was explained by 2 axes. The addition of a third axis did not improve the model substantially.

While the percentages of shared and unshared OTUs between the four quadrants for the entire dataset were similar (52.3% shared between 2 or more quadrants, and 47.7% unique, respectively), the majority of the total sequences (85.9%) were shared between at least two quadrants (as opposed to 14.1% unshared). This disparity between the percentage of shared sequences and the percentage of shared OTUs between the 4 quadrants argues that members with low abundance play a disproportionate role in the observed high level of beta diversity. To examine such assumption, each quadrant was divided into rare versus abundant OTUs, using various rarity (number of clones (n) = 1, n≤2, n≤5, and n≤10, or percentage abundance≤0.004%), and abundance (n≥10, and percentage abundance>1%) cutoffs. The numbers of OTUs in each of these groups for each quadrant and the total are shown in Table 3. For each of the rare and abundant OTU groups, various beta diversity estimates (as described above) were conducted. In general, Venn diagram displayed lower proportion of shared OTUs between rare members of the four communities when contrasted to the proportion of shared OTUs between abundant members of the community (Figure 2A–G). Various similarity indices (Table 2) showed higher beta diversity (i.e. lower similarity) between rare members of the community in the four quadrants, as opposed to the abundant fraction of the community, or the entire dataset of the four quadrants. Finally, non-metric multidimensional scaling plots using Bray-Curtis similarity indices of rare, abundant, and total datasets (Figure 3) showed that while various members belonging to the same quadrant were close to each other in ordination space, all abundant members from the 4 quadrants clustered very close together indicating that, regardless of the quadrant, abundant members are more similar to each other as opposed to rare members that showed more further apart positioning in ordination space giving further evidence to the observation that rare members of the community contribute more to β-diversity compared to abundant members.

**Table 3:** Number of OTUs_0.03_ identified for each quadrant at each of the rare and abundant species definitions used in this study.

Quadrant	n = 1	n≤2	n≤5	n≤10	n>10	a≤0.004%	a>1%
1	12140	14652	16589	17340	925	15286	7
2	11052	13618	15551	16335	953	14231	7
3	12526	14999	16785	17505	903	15566	6
4	9068	10730	12075	12632	634	10730	5
Total^a^	39062	44658	47581	48579	2203	46050	21

n: corresponds to the number of clones, a: corresponds to percentage abundance.

a Total OTUs correspond to the number of OTUs identified when reads from all quadrants are combined at the specified cutoff.

### Is the observed β diversity between rare members of the community due to inadequate sampling?

Rarefaction analysis for the number of OTUs observed in each quadrant studied (Figure S2) shows that our sampling is far from adequate to observe all species present in the spring. Since the probability of missing rare members of the community upon incomplete sampling is higher than the probability of missing more numerically abundant members of the community, it is conceivable that the observed β-diversity between the rare members of the community could be due to inadequate sampling. As such, it could be argued that the high beta diversity differences observed might not be truly due to spatial differences in these communities, and could primarily be mediated by inadequate sampling.

**Figure 4:**
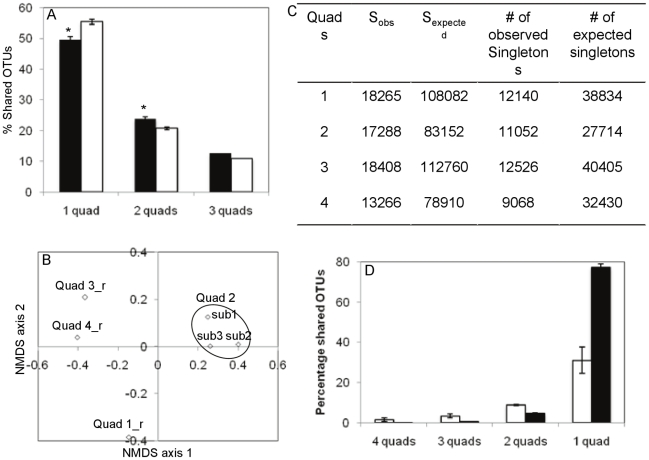
Effect of inadequate sampling on the observed beta diversity in Zodletone spring. A sub-sampling approach was implemented in which 3 subsamples from quadrant 2 (sub1, sub2, and sub3) were compared to three subsamples, each from a different quadrant (Quad 1_r, Quad 3_r, and Quad 4_r). (A) The percentage unshared OTUs, and the percentage shared OTUs between 2 quadrants, and 3 quadrants for the 3 subsamples from quadrant 2 versus the 3 subsamples from quadrants 1, 3, and 4 (* denotes that the percentage shared/unshared OTUs were significantly different between the 2 groups). Error bars represent standard deviations from 3 pairs of subsamples (2 quads) and 3 subsamples (1 quad). No error bars were obtained for the single 3-quads data point. Color-coding is as follows: the 3 subsamples from quadrant 2 (black), and the 3 subsamples from quadrants 1, 3, and 4 (white). (B) Non-metric multidimensional scaling plot for the 6 subsamples studied. Pair wise Bray-Curtis similarity indices were used to construct the plot. Stress value was close to 0 and most of the variation (77.8%) was explained by 2 axes. (C) Observed and expected number of species (S_obs_, and S_expected_, respectively) and singletons for each of the quadrants studied. The numbers were used to construct theoretical Venn diagrams as described in text. (D) The percentage shared OTUs between 4 quadrants, 3 quadrants, 2 quadrants, and the percentage unshared OTUs calculated from the obtained Venn diagram for n = 1 (shown in Figure 2A) (observed data), and the average percentages calculated from the theoretical Venn diagrams (predicted data). Error bars represent standard deviations from 4 data points for each of the observed and predicted unshared OTUs percentages (1 quad), 6 data point for the observed shared OTUs percentages and 4 data points for the predicted shared OTUs percentages between 2 quads, 4 data points for each of the observed and predicted shared OTUs percentages between 3 quads, 4 data points for the predicted shared OTUs percentages between 4 quads. No error bars were obtained for the single 4-quads observed shared OTUs percentage data point. Color-coding is as follows: Observed data (black), and predicted data (white).

To address such issues, we implemented a sub-sampling approach in which 3 subsamples from a single quadrant (quadrant 2) were compared to three subsamples, each from a different quadrant (subsamples from quadrants 1, 3, and 4). As such, if the beta diversity differences observed are truly spatial, then higher Beta diversity levels would be observed between subsamples from quadrants 1, 3, and 4 as opposed to the subsamples from quadrant 2. We created 3 random subsamples (28,000 sequences each) from our largest dataset (quadrant 2) and treated the subsamples as separate datasets. We also randomly picked a 28,000-sequence subsample from each of the other 3 quadrants. We hypothesized that since the 3 random sub-samples from quadrant 2 have no spatial separation, then they should be more similar to one another when compared to the 3 random sub-samples from the other 3 quadrants. Moreover, beta diversity observed between the subsamples of quadrant 2 would also act as a baseline for comparison where any significantly different β-diversity beyond that baseline could possibly be a true and not a random difference. Figure 4A shows that the 3 sub-samples from quadrant 2 have 12.6% of the total OTUs shared between the 3 sub-samples, 23.8±0.55% of the total OTUs shared between 2 subsamples, and 49.6±0.93% of the total OTUs unshared. Student t-test showed that subsamples from quadrants 1, 3, and 4 had significantly less shared OTUs percentage (10.8% of the total OTUs shared between the 3 sub-samples, 20.7±0.46% of the total OTUs shared between 2 subsamples), and significantly more unshared OTUs (55.4±0.93% of the total OTUs unshared) (p = 0.0016). Non-metric multidimensional scaling using Bray-Curtis similarity indices clustered the 3 sub-samples from quadrant 2 close to each other in ordination space, while the 3 subsamples from quadrants 1, 3, and 4 clustered further apart in ordination space from one another as well as from the subsamples of quadrant 2 (Figure 4B). The above results argue that the observed differences between quadrants are not random and could possibly be due to their spatial separation.

To examine the possibility that the observed β-diversity between the rare members of the community is due to inadequate sampling, we used the best-fit parametric estimator (mixture of 2 exponentials-mixed Poisson) to estimate the species richness, as well as the predicted distribution patterns within each quadrant, using the criteria previously outlined [37], [38]. Using this approach, we estimated the number of expected singletons in each quadrant had a complete census of the microbial community been achieved. Figure 4C shows the numbers of observed and expected OTUs as well as singleton OTUs in each of the 4 quadrants studied. In theory, if we have a pool of the expected number of singletons (e.g. 38,834 singletons in quadrant 1) and we sampled the number of observed singletons (e.g. 12140 singletons in quadrant 1) from that pool with replacement 4 times (i.e. four-12140 random draws from a pool of 38,834 singletons), we should be able to construct a theoretical Venn diagram and determine the expected percentages of singletons shared between 4 samples, between 3 samples, between 2 samples, and the expected percentage of unshared singletons. A theoretical Venn diagram was constructed for each of the 4 quadrants and the average percentages of shared and unshared OTUs were calculated. Those are the predicted percentages of shared and unshared OTUs. We used these theoretical values as a baseline for comparison to our actual (observed) percentages of singletons shared between 4 samples, between 3 samples, between 2 samples, and the average percentage of unshared singletons. Since the 4 random draws to construct the theoretical Venn diagram (and hence calculate the predicted shared and unshared percentages) come from the same pool, then any significant difference from the predicted values should be regarded as true difference and not due to inadequate sampling. Figure 4D shows the average predicted % of shared and unshared singletons and the corresponding observed percentages (shown in Table S3 for n = 1). The observed unshared singletons percentage was significantly higher (77.3±1.78% compared to a predicted value of 31.1±6.64%), and the observed shared singletons percentages were significantly lower (0.1%, 0.59±0.04%, and 4.7±0.19% compared to predicted values of 1.54%, 3.39±1%, and 8.73±0.23%) compared to the corresponding predicted values. Therefore we can conclude that, while inadequate sampling could contribute to observed beta diversities within rare biosphere of highly diverse communities, the observed β-diversity in Zodletone spring quadrants could not be fully explained by inadequate sampling.

### What fraction of the rare biosphere is mostly responsible for the observed Beta diversity?

As previously suggested [20], members of the rare biosphere are a heterogeneous mix of microorganisms with various phylogenetic affiliations and relationships to other members of the community. Broadly, members of the rare biosphere could be: novel and unique i.e. belong to novel, previously un-encountered phylogenetic lineages and have no close relatives within more abundant members of the community, unique but non-novel, i.e. have no similarity to any abundant members of the community but belong to previously described phylogenetic lineages, and non-novel and non-unique i.e. having relatives within the most abundant members of the community.

We sought to compare levels of beta diversities of each of these three groups to pinpoint the members of the rare biosphere that contribute the most to the observed β-diversity (i.e. Across the four datasets; which specific subgroup of the rare biosphere varies the most between samples?). We divided our rare biosphere (arbitrarily defined as n≤5) into the 3 subgroups according to the criteria described in Materials and Methods. In general, the majority of rare clones were classified as non-unique non-novel (NUNN 90.5%), followed by the unique non-novel members (UNN 5%) then the unique novel members (UN 4.5%). Table 4 shows beta diversity measures for each of the subgroups of the rare biosphere as well as for the total rare biosphere defined at n≤5 studied across the 4 quadrants. Analysis of variance showed that for all the beta diversity measures, the 3 subgroups of the rare biosphere as well as the total rare biosphere (n≤5) were significantly different (p<0.0001). To further pinpoint the member of the rare biosphere responsible for such significant difference in beta diversity measures, pair wise t-tests between all possible pairs (NUNN-UN, NUNN-UNN, NUNN-total, UN-UNN, UN-total, and UNN-total) were carried out. Results showed that the unique non-novel members were the most responsible for that significant difference. Unique non-novel members had significantly higher percentage of unshared OTUs and significantly lower percentage of shared OTUs compared to the total rare biosphere as well as the other 2 rare subgroups with p-values<0.0083 (Bonferonni-corrected p-value for the number of pair-wise comparisons).

**Table 4:** Beta diversity measures for the rare biosphere sub-groups across the 4 quadrants compared to the total rare biosphere (n≤5).

Rare sub-group^a^	Percentage of OTUs shared between				Pair wise similarity indices^c^			
	4 quads	3 quads^b^	2 quads^b^	1 quad^b^	Abundance-based		Theta (Smith)	Theta (Yue & Clayton)
					Jaccard	Sorensen		
NUNN	2.1	4.5±0.12	12.9±0.37	57.2±3.2	0.3±0.01	0.47±0.01	0.17±0.004	0.18±0.005
UN	2.3	4.7±0.52	12.1±0.87	63±2.12	0.3±0.05	0.48±0.06	0.18±0.008	0.2±0.007
UNN	1.1	2.6±0.18	8.2±0.58	71±2.16	0.23±0.02	0.37±0.03	0.11±0.006	0.12±0.004
Total	2.3	4.7±0.09	13.2±0.29	56.9±3.12	0.32±0.008	0.48±0.01	0.18±0.005	0.18±0.005

a NUNN are the non-unique non-novel members, UN are the unique novel members, UNN are the unique non-novel members, total correspond to the total rare biosphere defined at the empirical cutoff of n≤5.

b Numbers are averages ± standard deviations of shared/unshared OTUs percentages for 4 data points (1 quad, and 3 quads) and 6 data points (2 quads). The percentages were calculated by dividing the number of shared/unshared OTUs between “x” quadrants by the total number of observed OTUs in those “x” quadrants. The number of shared OTUs between 2 quadrants also includes the number of OTUs that these 2 quadrants share with either one or two more quadrants. The number of shared OTUs between 3 quadrants also includes the number of OTUs that these 3 quadrants share with the fourth quadrant.

c All similarity indices values are averages ± standard deviations of 6 pair wise comparisons. We only chose those similarity indices that use the relative abundance data (instead of the abundance or the presence-absence data) due to the difference in sample sizes of the 4 groups compared.

## Discussion

In this study, we examined the bacterial diversity in an anaerobic sulfur- and sulfide-rich spring in southwestern Oklahoma. We used 292,130 high quality 16S rRNA gene sequences, distributed between four samples to study patterns of fine scale β-diversity within the community. While comparative diversity between multiple communities has been extensively studied using culture-based [39], [40], [41], [42], and culture independent [6], [7], [12], [14], [18] surveys, most of these studies have focused on elucidating the effect of specific measurable factor(s) on microbial community structure e.g. along gradients of various length scales, from cm [43] to global [44], [45] scales. This work is significant since it examines whether fine scale beta diversity exists in seemingly identical samples in a highly diverse ecosystem, and what proportion of the community is responsible for such differences. The most important findings of this study is the fact that high beta diversity is detected within spatially (1 mm) separated samples at Zodletone spring source sediments, with rare members of the community primarily responsible for such diversity. This issue should be taken into account when 1: interpreting results of comparative pyrosequencing surveys, especially those comparing rare biosphere across various ecosystems; 2: Assessing the overall microbial diversity (gamma diversity) within highly diverse ecosystems; and 3. Planning for metagenomic surveys of such environments.

Our study shows that phylum-level diversity profile from a specific habitat is highly reproducible (Figure 1), and hence results from pyrosequencing surveys should be viewed as accurately representing phylum level diversity within the entire habitat. On the other hand, our results indicate that the notion that species level diversity profile of an ecosystem could accurately be described in a single pyrosequencing survey should be interpreted with caution. Profiles of the abundant members of a community appear to be reproducible and highly similar between spatially separated samples. On the other hand, studies comparing the rare biosphere across different ecosystems should be handled with caution, since the composition of the rare biosphere within a specific ecosystem appear to vary significantly between seemingly identical samples, and hence a single sample is not truly representative of the rare biosphere of the entire ecosystem [9]. In our dataset, since only 23–47% on average of the rare OTUs (at rarity cutoffs of n = 1 and n = 10) were shared, then considering the rare OTUs from a single quadrants as the signature OTUs of the community and using such data in comparing rare Zodletone OTUs to other OTUs is highly problematic.

In addition to the implications on interpreting comparative pyrosequencing studies, the detected high levels of beta diversity on a fine scale within a complex ecosystem should be taken into consideration upon estimating the overall diversity (gamma diversity) within a specific ecosystem. Efforts to gauge diversity and species richness in complex habitats (e.g. soil) have been an area of intense research. However, in most cases, the starting point of reported analysis would be a single clone library or a pyrosequencing dataset derived from a single PCR reaction [37], [46], [47]. As such, these admirable efforts provide estimates on the diversity within a fraction of the habitat studied i.e. from the DNA included in the PCR reaction, which is only a fraction of the DNA isolated from a single sample (e.g. 0.5 grams of soil). By no means such numbers would be considered representative of the species richness and diversity of the entire habitat to be studied. Therefore estimates of microbial gamma diversity of a specific habitat should take fine scale beta diversity patterns into account rather than relying solely on alpha diversity information.

In addition to estimates of species richness and overall diversity, culture-independent surveys are often used as a starting point for planning metagenomic surveys of entire communities. Estimates for sequencing effort required for obtaining high (e.g. 90%) coverage within such habitats have been reported [48], [49], but have often been based on single 16S rRNA gene datasets. We argue that ignoring beta diversity patterns could lead to vast underestimation of sequencing efforts required to achieve the desirable coverage. This is especially important since recent and anticipated advances in sequencing technologies [50], [51], bioinformatics programs and pipelines [52], [53], [54], improvements in existing sequencing technologies [55] have triggered efforts for conducting coverage-oriented metagenomic surveys aiming at complete sequencing of an entire ecosystem [56], [57]. We argue that upon taking beta diversity patterns into account, the strict notion of complete sequencing of an entire ecosystem (e.g. soil) is a near impossibility. Rather, a more realistic, yet-still extremely ambitious and desirable goal would be the complete sequencing of abundant, fine-scale, shared members of the community since obtaining coverage of these members are achievable. Also, in addition to the huge additional sequencing efforts required to chase the long tail end of the extremely alpha and beta diverse rare biosphere, genomes derived from cells that are encountered only once in a single gram of soil could be lost during sample preparation and titration phases of the project, and hence completely missed in such census.

Finally the results reinforce the nation that the rare biosphere is a near inexhaustible supply of genomic novelty. In Zodletone spring, a significant proportion of sequences belonged to novel and unique lineages (3804 sequences). The fact that a high level of beta diversity (only 37% shared between 2 or more quadrants) was encountered in this group, argues that the potential for discovering novel lineages within a specific habitat could not be adequately assessed using a single sample, an issue that could greatly expand the inventories of novel lineages within the earth biosphere than previously implied by single dataset survey of complex habitats.

## Supporting Information

Figure S1 (A) Zodletone spring source. Anaerobic, and sulfide-rich black viscous sediments covered by an anoxic, sulfide-rich water column were sampled according to the technique in (B). The grid used for obtaining samples is composed of 1 inch2 grids 1-mm thick. Four quadrants of sediments were used for DNA extraction such that all 4 samples are 1 mm apart from each other.
(1.89 MB TIF)

Figure S2 Rarefaction curves of the observed number of operational taxonomic units defined at 97% sequence similarity (OTUs0.03) for each of the quadrants studied. Color-coding is as follows: quadrant 1 (red), quadrant 2 (black), quadrant 3 (green), and quadrant 4 (blue).
(0.31 MB TIF)

Text S1 Sequences from quadrant 1.
(4.64 MB ZIP)

Text S2 Sequences from quadrant 2.
(4.96 MB ZIP)

Text S3 Sequences from quadrant 3.
(4.48 MB ZIP)

Text S4 Sequences from quadrant 4.
(3.10 MB ZIP)

Table S1 Effect of quality filtering on the number of reads obtained for each quadrant.
(0.06 MB DOCX)

Table S2 Phyla percentage abundance in the 4 quadrants studied as well as in the total.
(0.11 MB DOCX)

Table S3 Percentage of shared and unique OTUs and clones between the 4 quadrants studied at all the rare and abundant empirical cutoffs as well as for the totals.
(0.11 MB DOCX)
