# Supplementary figures and images for: Fine-Scale Bacterial Beta Diversity within a Complex Ecosystem (Zodletone Spring, OK, USA): The Role of the Rare Biosphere
Source: PLoS One. 2010 Aug 26;5(8):e12414. doi: 10.1371/journal.pone.0012414 (PMC2932559; doi:10.1371/journal.pone.0012414)

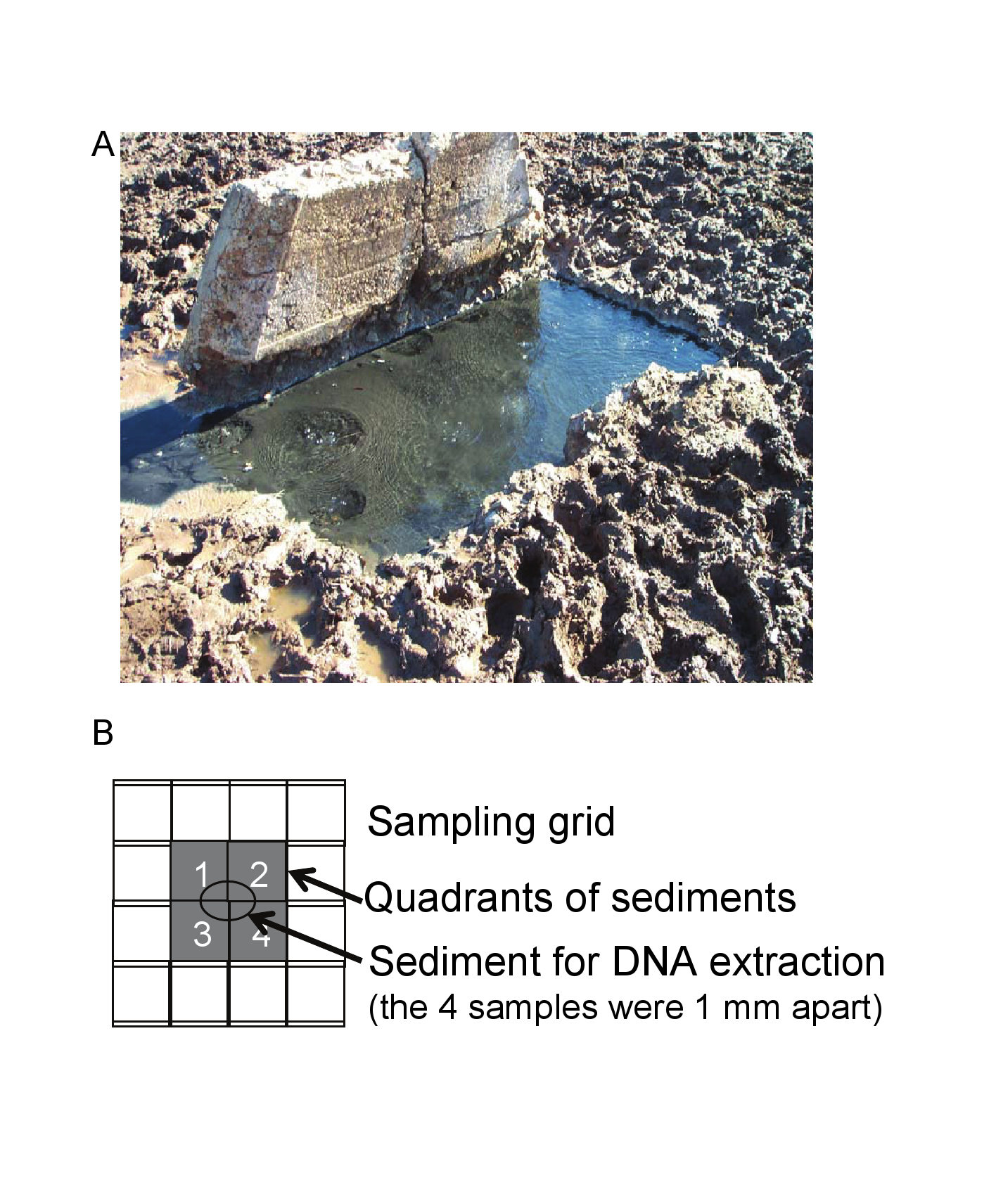

Supplement: Figure S1 — (A) Zodletone spring source. Anaerobic, and sulfide-rich black viscous sediments covered by an anoxic, sulfide-rich water column were sampled according to the technique in (B). The grid used for obtaining samples is composed of 1 inch2 grids 1-mm thick. Four quadrants of sediments were used for DNA extraction such that all 4 samples are 1 mm apart from each other. (1.89 MB TIF) [file pone.0012414.s001.tif]

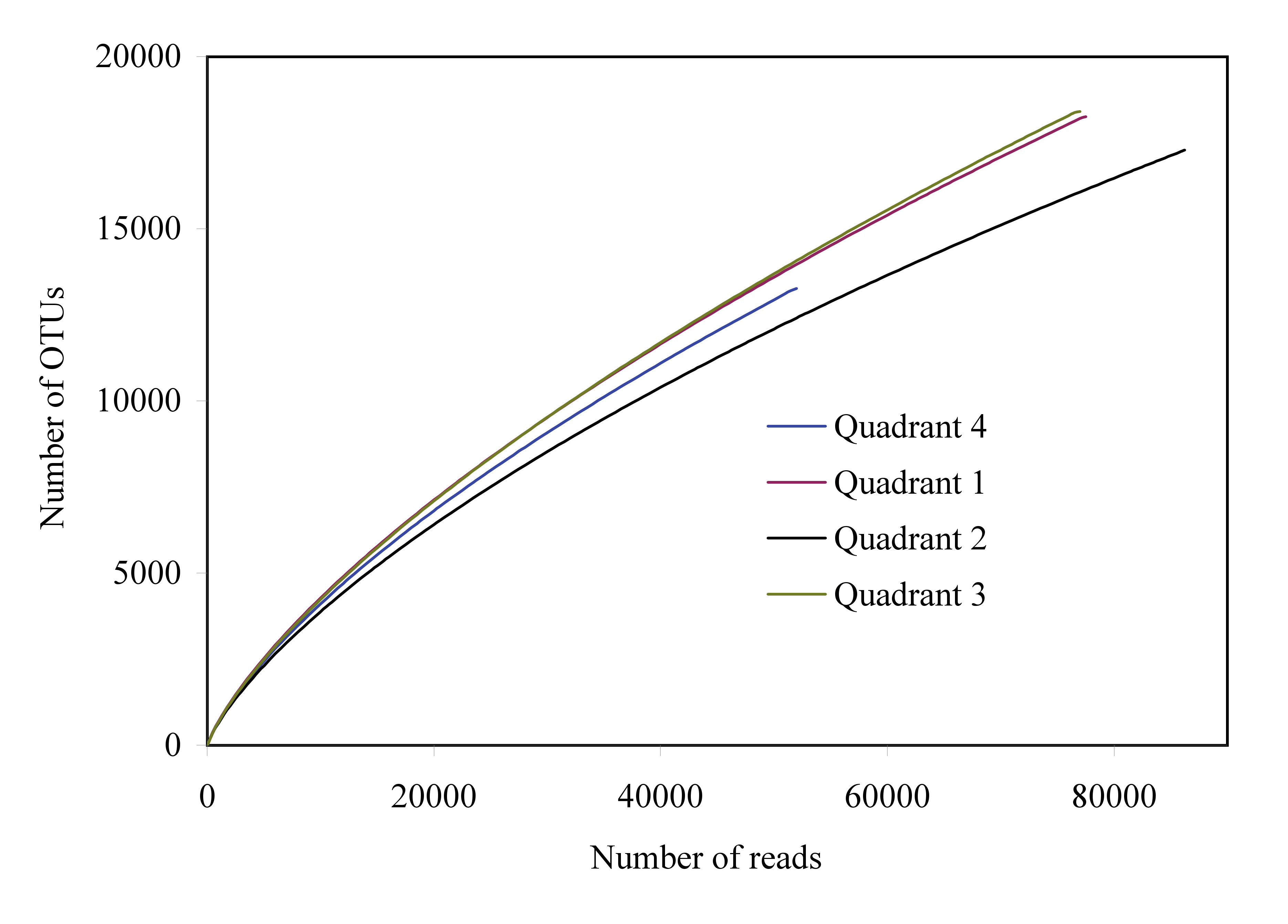

Supplement: Figure S2 — Rarefaction curves of the observed number of operational taxonomic units defined at 97% sequence similarity (OTUs0.03) for each of the quadrants studied. Color-coding is as follows: quadrant 1 (red), quadrant 2 (black), quadrant 3 (green), and quadrant 4 (blue). (0.31 MB TIF) [file pone.0012414.s002.tif]
