# Supplementary material for: Fine-Scale Bacterial Beta Diversity within a Complex Ecosystem (Zodletone Spring, OK, USA): The Role of the Rare Biosphere
Source: PLoS One. 2010 Aug 26;5(8):e12414. doi: 10.1371/journal.pone.0012414 (PMC2932559; doi:10.1371/journal.pone.0012414)
Supplement: Table S1 — Effect of quality filtering on the number of reads obtained for each quadrant. (0.06 MB DOCX) [file pone.0012414.s007.docx]

Table S1. Effect of quality filtering on the number of reads obtained for each quadrant^a^.

| Quadrant | Number of initial reads | Reads after removing low-quality reads (reads with average quality <25) | Reads after removing reads with ambiguous bases | Reads after removing reads with wrong primer sequence | Reads after removing short reads (<80 bp) and reads with homopolymers >8 |
| --- | --- | --- | --- | --- | --- |
| 1 | 92179 | 91127 (98.9) | 88127 (95.6) | 83885 (91) | 77361 (83.9) |
| 2 | 97747 | 97351 (99.6) | 95140 (97.3) | 90985 (93.1) | 86240 (88.2) |
| 3 | 92796 | 91657 (98.8) | 88373 (95.2) | 84851 (91.4) | 76693 (82.6) |
| 4 | 65364 | 63327 (96.9) | 62244 (95.2) | 59616 (91.2) | 51836 (79.3) |

a: Numbers in parentheses are percentage
