# Supplementary material for: Fine-Scale Bacterial Beta Diversity within a Complex Ecosystem (Zodletone Spring, OK, USA): The Role of the Rare Biosphere
Source: PLoS One. 2010 Aug 26;5(8):e12414. doi: 10.1371/journal.pone.0012414 (PMC2932559; doi:10.1371/journal.pone.0012414)
Supplement: Table S2 — Phyla percentage abundance in the 4 quadrants studied as well as in the total. (0.11 MB DOCX) [file pone.0012414.s008.docx]

Table S2. Phyla percentage abundance in the 4 quadrants studied as well as in the total.

| Phylum | Percentage abundance in | | | | |
| --- | --- | --- | --- | --- | --- |
|  | Quad 1 | Quad 2 | Quad 3 | Quad 4 | Total |
| Unclassified^a^ | 29.42 | 32.97 | 32.71 | 32.97 | 31.96 |
| *Bacteroidetes* | 26.31 | 27.05 | 25.02 | 24.57 | 25.88 |
| *Firmicutes* | 12.89 | 12.6 | 14.19 | 12.63 | 13.1 |
| *Spirochaetes* | 4.95 | 6.2 | 5.08 | 4.88 | 5.34 |
| δ-*Proteobacteria* | 5.07 | 4.76 | 5.61 | 5.6 | 5.22 |
| α-*Proteobacteria* | 4.38 | 3.35 | 3.3 | 3.35 | 3.61 |
| γ-*Proteobacteria* | 3.37 | 2.9 | 3.27 | 2.87 | 3.12 |
| *Chloroflexi* | 2.62 | 2.26 | 2.73 | 3.23 | 2.65 |
| β-*Proteobacteria* | 3.56 | 1.79 | 1.9 | 1.9 | 2.31 |
| *Actinobacteria* | 1.49 | 1.22 | 1.13 | 1.28 | 1.28 |
| ε-*Proteobacteria* | 1.9 | 0.79 | 1.02 | 1.14 | 1.21 |
| *Acidobacteria* | 1.26 | 1.07 | 1.13 | 1.11 | 1.14 |
| *Chlorobi* | 0.33 | 0.575 | 0.519 | 1.74 | 0.702 |
| *Gemmatimonadetes* | 0.398 | 0.372 | 0.406 | 0.394 | 0.392 |
| *Verrucomicrobia* | 0.301 | 0.333 | 0.293 | 0.361 | 0.319 |
| *Fusobacteria* | 0.27 | 0.399 | 0.289 | 0.278 | 0.315 |
| OP8 | 0.234 | 0.158 | 0.158 | 0.311 | 0.205 |
| WS3 | 0.178 | 0.186 | 0.166 | 0.197 | 0.18 |
| *Cyanobacteria* | 0.081 | 0.206 | 0.132 | 0.12 | 0.138 |
| OP9_JS1 | 0.138 | 0.077 | 0.102 | 0.201 | 0.122 |
| TM7 | 0.137 | 0.099 | 0.098 | 0.077 | 0.105 |
| *Thermotogae* | 0.092 | 0.09 | 0.085 | 0.108 | 0.092 |
| GN02 | 0.087 | 0.088 | 0.074 | 0.05 | 0.077 |
| *Nitrospirae* | 0.08 | 0.058 | 0.074 | 0.091 | 0.074 |
| *Planctomycetes* | 0.057 | 0.06 | 0.063 | 0.093 | 0.066 |
| TM6 | 0.062 | 0.048 | 0.06 | 0.06 | 0.057 |
| *Synergistetes* | 0.03 | 0.043 | 0.08 | 0.052 | 0.051 |
| SPAM | 0.062 | 0.027 | 0.039 | 0.056 | 0.045 |
| OP1 | 0.021 | 0.023 | 0.026 | 0.052 | 0.028 |
| *Fibrobacteres* | 0.009 | 0.021 | 0.03 | 0.008 | 0.018 |
| OP10 | 0.018 | 0.008 | 0.014 | 0.039 | 0.018 |
| *Caldithrix*_KSB1 | 0.023 | 0.019 | 0.02 | 0.008 | 0.018 |
| WCHB1-27 | 0.017 | 0.021 | 0.005 | 0.015 | 0.015 |
| ABY1_OD1 | 0.012 | 0.007 | 0.02 | 0.019 | 0.014 |
| Marine_group_A | 0.008 | 0.012 | 0.01 | 0.035 | 0.014 |
| OP11 | 0.012 | 0.006 | 0.022 | 0.014 | 0.013 |
| OP3 | 0.008 | 0.019 | 0.012 | 0.014 | 0.013 |
| *Thermi* | 0.005 | 0.017 | 0.013 | 0.017 | 0.013 |
| WS6 | 0.009 | 0.003 | 0.023 | 0.021 | 0.013 |
| KSB3_GN06 | 0.019 | 0.014 | 0 | 0 | 0.009 |
| NC10 | 0.009 | 0.006 | 0.009 | 0.008 | 0.008 |
| ZB3 | 0.014 | 0 | 0.008 | 0.014 | 0.008 |
| *Elusimicrobia*_TG1 | 0.009 | 0.007 | 0.007 | 0.006 | 0.007 |
| AD3 | 0.005 | 0.003 | 0.003 | 0.008 | 0.004 |
| SM2F11 | 0.003 | 0.003 | 0.007 | 0.006 | 0.004 |
| 49S1_2B_6 | 0.003 | 0.005 | 0.001 | 0.004 | 0.003 |
| *Haloanaerobiales* | 0.003 | 0 | 0.004 | 0 | 0.002 |
| NKB19 | 0.003 | 0 | 0.003 | 0.002 | 0.002 |
| SC3 | 0.001 | 0.001 | 0.003 | 0.002 | 0.002 |
| *Lentisphaerae* | 0.001 | 0.001 | 0.005 | 0 | 0.002 |
| SC4 | 0.004 | 0.001 | 0.003 | 0 | 0.002 |
| CV51 | 0.001 | 0.001 | 0.003 | 0.006 | 0.002 |
| *Entotheonella* | 0.004 | 0.002 | 0.001 | 0.002 | 0.002 |
| BRC1 | 0.001 | 0 | 0.003 | 0 | 0.001 |
| MBMPE71 | 0.003 | 0 | 0.001 | 0 | 0.001 |
| VHS-B5-50 | 0.003 | 0.001 | 0.001 | 0 | 0.001 |
| *Deferribacteres* | 0.001 | 0 | 0 | 0.002 | 0.0007 |
| GAL15 | 0.001 | 0 | 0.001 | 0 | 0.0007 |
| SR1 | 0 | 0 | 0 | 0.004 | 0.0007 |
| *Aquificae* | 0 | 0 | 0 | 0.002 | 0.0003 |
| *Natronoanaerobium* | 0 | 0 | 0 | 0.002 | 0.0003 |
| P9X2b3A04 | 0 | 0.001 | 0 | 0 | 0.0003 |
| *Sulfobacilli* | 0.001 | 0 | 0 | 0 | 0.0003 |
| *Thermoanaerobacteria* | 0 | 0 | 0 | 0.002 | 0.0003 |
| WPS-2 | 0.001 | 0 | 0 | 0 | 0.0003 |

a: Unclassified sequences with less than 85% similarity to their closest relative in Greengenes database
