# Supplementary material for: Fine-Scale Bacterial Beta Diversity within a Complex Ecosystem (Zodletone Spring, OK, USA): The Role of the Rare Biosphere
Source: PLoS One. 2010 Aug 26;5(8):e12414. doi: 10.1371/journal.pone.0012414 (PMC2932559; doi:10.1371/journal.pone.0012414)
Supplement: Table S3 — Percentage of shared and unique OTUs and clones between the 4 quadrants studied at all the rare and abundant empirical cutoffs as well as for the totals. (0.11 MB DOCX) [file pone.0012414.s009.docx]

Table S3. Percentage of shared and unique OTUs and clones between the 4 quadrants studied at all the rare and abundant empirical cutoffs as well as for the totals.

| Cutoff^a^ | Average % Shared between 2 quadrants^b^ | | Average % Shared between 3 quadrants^b^ | | Percentage shared between 4 quadrants^b^ | | Average % Unshared^b^ | | Average total % shared between quadrants^c^ | |
| --- | --- | --- | --- | --- | --- | --- | --- | --- | --- | --- |
|  | OTUs | Sequences | OTUs | Sequences | OTUs | Sequences | OTUs | Sequences | OTUs | Sequences |
| Total | 21.4 | 79.1 | 11.3 | 74.6 | 7.6 | 72.3 | 47.7 | 14.1 | 52.3 | 85.9 |
| n=1 | 4.71 | 9 | 0.6 | 1.6 | 0.1 | 0.4 | 77.3 | 77.3 | 22.7 | 22.7 |
| n≤2 | 8.2 | 16.4 | 1.8 | 5.3 | 0.6 | 2.1 | 67.5 | 64.2 | 32.5 | 35.8 |
| n≤5 | 13.2 | 29.9 | 4.7 | 15.9 | 2.3 | 9.9 | 56.9 | 47.7 | 43.1 | 52.3 |
| n≤10 | 16.2 | 40.8 | 7 | 27.3 | 4 | 20.6 | 53.1 | 38.5 | 46.9 | 61.5 |
| a≤0.004% | 10 | 21 | 2.8 | 8.4 | 1.1 | 4.1 | 62.4 | 57.7 | 37.6 | 42.3 |
| n>10 | 6.3 | 91.9 | 8.7 | 89.1 | 36 | 87.3 | 12.2 | 3.8 | 87.8 | 96.2 |
| a>1% | 64.3 | 77.2 | 52.7 | 71.8 | 47.6 | 71.6 | 10.8 | 11.1 | 89.2 | 88.9 |

a: The empirical cutoff used to define rare and abundant members of the community. n: corresponds to the number of clones, a: corresponds to percentage abundance.

b: Numbers are averages ± standard deviations of shared/unshared OTUs (or sequences) percentages for 4 data points (1 quad, and 3 quads) and 6 data points (2 quads). The percentages were calculated by dividing the number of shared/unshared OTUs (or sequences) between “x” quadrants by the total number of observed OTUs (or sequences) in those “x” quadrants. The number of shared OTUs (or sequences) between 2 quadrants also includes the number of OTUs (or sequences) that these 2 quadrants share with either one or two more quadrants. The number of shared OTUs (or sequences) between 3 quadrants also includes the number of OTUs (or sequences) that these 3 quadrants share with the fourth quadrant.

c: the total % shared OTUs (or sequences) were simply calculated as 100-total % unshared OTUs (or sequences)
